# The Effects of Cogging Torque Reduction in Axial Flux Machines

**DOI:** 10.3390/mi12030323

**Published:** 2021-03-19

**Authors:** Samuel Mengesha, Shailendra Rajput, Simon Lineykin, Moshe Averbukh

**Affiliations:** 1 Department of Electric and Electronic Engineering, Ariel University, Ariel 40700, Israel; SAMY1604@hotmail.com; 2 Department of Mechanic Engineering and Mechatronics, Ariel University, Ariel 40700, Israel; simonl@ariel.ac.il

**Keywords:** cogging effect, axial flux machine, torque, magnetic flux

## Abstract

An axial flux permanent magnet single-rotor generator has good potential in various applications that require high efficiency, prolonged service life, as well as low mass and dimensions. However, the effect of cogging torque diminishes generator efficiency and flexibility of functionality. The effect of cogging torque arises because of a small air gap between the stator teeth and the rotor. In this article, we suggest that shifting the opposite teeth of the stator to the optimal angle can reduce the effect of cogging torque. A special axial flux permanent magnet generator is developed to choose the optimal disposition of the permanent magnet and stator teeth in the frame. The impact of the optimal angle on the cogging torque, output power, and generator efficiency is investigated. This analytical study with experimental testing proves that the optimal angle between opposite teeth can significantly decrease cogging torque and improve output power and efficiency. The results show that cogging torque decreases significantly (4–5 times) at an optimal angle of 7.5° as compared with that of other angles, although magnetic flux and output power decline slightly but efficiency increases.

## 1. Introduction

Recently, axial flux permanent magnet (AFPM) electrical machines have represented the most potential class of energy transforming appliances, which provide the best power/weight ratio and excellent efficiency for different applications [1,2,3,4]. The absence of rotor winding due to strong NeFe composite permanent magnets (PMs) and the optimal use of stator–rotor surface enables high torque at low velocities, good cooling possibilities, and high energy efficiency [5].

However, the use of PMs produces the effect of cogging torque, which arises due to the interactions between the PMs and stator slots. The tendency of PM rotors to align in a few stable positions results in an alternating pulsating force (also known as cogging torque); cogging torque does not contribute to the average torque [4,6,7]. The effect of cogging torque decreases the accuracy of the motor positioning at low speed, produces additional friction resistance, and to some extent, reduces the output power if the axial flux machine is applied as an electrical generator [8,9,10]. Cogging torque reduction is an important design goal in AFPM machines. It exists even if there is no current flowing in the stator windings (the machine is not excited) [4]. Several analytical studies have been conducted to explain and describe the effects of cogging torque [11,12,13,14,15].

Previously, several methods have been proposed and analyzed for reduce the effect of cogging torque [16,17,18,19,20,21]. Some studies have suggested an optimal design of axial flux machines to diminish the effect of cogging torque [22,23,24,25]. Optimal shaping of the rotor poles was proposed by Sikder et al. [21]. Optimization of the air gap between the stator and disc rotor was suggested by Saygin et al. [22]. Design parameters that can have an effect includes geometrical dimensions of teeth, number of teeth, and the angle between teeth [23]. Skewing of PMs or stator slots was suggested in [24,25]. It has been pointed out that the displacement between stator teeth is more effective for diminishing the cogging vs. skewing of slots or magnets. In a study by [25], the design of a machine based on a single stator frame was proposed. However, the structure’s design did not alter the shifting between opposite teeth during machine exploitation, which restricted the potential ability of the design. Studies have shown that multi-objective shape optimization [26] and single-objective optimization [27] can help to diminish the influence of the cogging torque effect. Numerous studies have been dedicated to experimentally studying reduction of cogging torque effect [28,29,30]. The emphasis on a no-load mode of an axial flux machine [28] has resulted in cogging torque reduction. Another study tested core losses and their influences on cogging torque [29]. In [30], the authors suggested asymmetry of the magnet arrangement and experimentally studied its influence on cogging torque. To the best of our knowledge, most previous work has been dedicated to the theoretical or experimental analysis of the effect of cogging torque in a narrow range of machine functionalities.

In this study, we provide a comprehensive experimental analysis of the cogging effect in a machine with a double-stator design. The torque deviations because of the cogging effect are studied through the rotor and stator rotation angle. The effect of the angle between opposite double-stator teeth on the cogging magnitudes and the output power is also studied.

## 2. Design Axial Flux Double-Stator Permanent Magnet (PM) Generator

We experimentally tested and analyzed a specially developed axial flux double-stator generator with PMs attached in the rotor. There were several reasons for the choice of this type of generator design. First, the magnetic flux penetrating the rotor placed inside two opposite halves of stators is higher than that in a generator with a double rotor; thus, it can effectively work in lower rotating velocities and produce more power. Second, a single rotor includes half of the PM segments as compared with a double-rotor frame; this circumstance decreases the cost of the machine, since the PMs determine a significant portion of the expenses. The third reason is related to cooling possibilities; the main heat flux is produced in the winding part of the machine, which is the stators located outside of a disk rotor equipped with permanent magnets, and therefore the ventilation cooling can be more efficient.

### 2.1. Prototype Design

The stator cores were made using cold-rolled grain-oriented silicon steel that was insulated with a special heat resistant coating. The material was designed to substantially reduce core losses and to produce certain magnetic properties and high permeability. The thickness of the core laminate was 0.05 mm, with a permeability of $\mu_{r}\;=\;40,000$, and an inner diameter of 43 mm and an outer diameter of 60 mm. The development and design of the stator core was based on the following merits:

i. Stator teeth should consider the dimensions of the PMs.
ii. The teeth dimensions should induce a maximal amount of torque between the stators and the PMs. For this aim, the teeth width should be slightly narrower than the width of a PM.
iii. The stator teeth shoes were made with the optimal dimensions for absorbing maximum magnetic flux.

**Figure 1:**
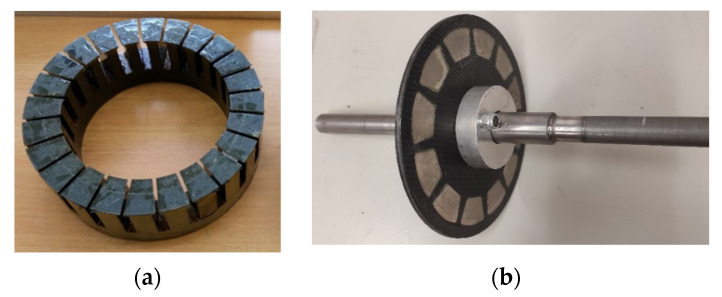
(**a**) Stator post CNC wire cutting; (**b**) Rotor and axle post assembly with PMs.

In each stator, 24 slots were created with a width of 6 mm and a height of 25 mm for each tooth. A CNC wire cutting machine was used due to the required complex geometry of the stators (Figure 1a). Each stator tooth is insulated with high temperature anti-static tape and wrapped with 70 turns of copper wire with a diameter of 0.5 mm. This appliance is a 12-pole machine configuration due to voltage inducing and cogging torque reduction considerations. The PM materials were chosen based on the following merits:

i. High magnetic flux density relative to weight and size;
ii. Temperature resistant;
iii. Availability and costs;
iv. Stator core size compatibility.

The PMs chosen for the system were neodymium (N45M) magnets with a composition of $Nd_{2}Fe_{14}B$. Such types of PMs are resistant to temperatures up to 90 °C. The system contained 12 magnet poles. The configuration of the rotor was based on the following requirements:

i. Non-metallic isolating material to support (frame) the PMs and eliminate loss due to conductivity of the backing material;
ii. An even number of PMs;
iii. Minimal thickness to reduce the air gap between the stators and the rotor;
iv. Reliable and strong enough to be machined while maintaining tolerances.

A 12-layer carbon fiber fabric reinforced with epoxy composite material (3 mm thickness) was chosen for the rotor. The material was cut using a CNC machine and attached to an iron axle using screws, shims, and attach plates for precise locating with an even distance from both stators to ensure an air gap of 0.5 mm (Figure 1b).

**Figure 2:**
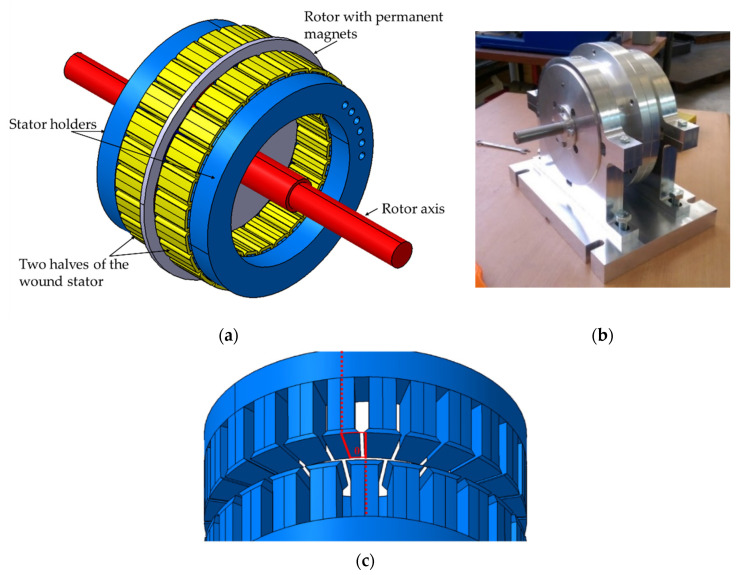
(**a**) Internal design of axial flux permanent magnet (AFPM); (**b**) AFPM generator yoke; (**c**) Relative angle between two halves of a stator.

**Figure 3:**
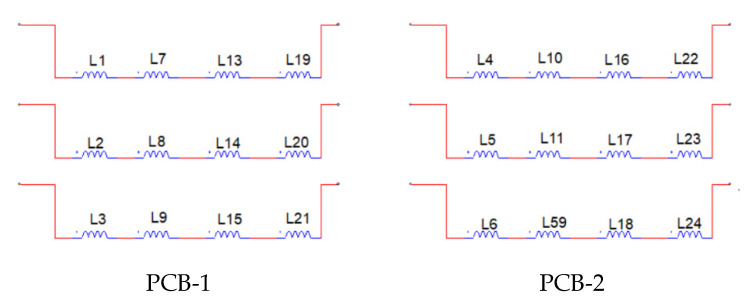
Stator coil connection arrangement for each stator.

Figure 2 demonstrates the internal design of AFPM, generator yoke and relative angle between two halves of a stator. Carbon fiber composites have a high strength to weight ratio and have a stiffness that is many times that of mild steel with the same thickness, while having a quarter of the weight. The axle was made of steel and manufactured to ensure the centering of the rotor between the stators. The yoke (body) of the generator was used as a frame and attached the system to the custom-built frame. The main requirements for the yoke were non-magnetic properties and strong enough to withstand axial forces and vibrations. Paramagnetic T6061 aluminum material was chosen for this purpose. The yoke’s stator-mating surface was equipped with a locating pin used for the stators’ pole-to-pole adjustment. The stator terminals of the 24 coils (single phase) were paired to groups of four coils (each start contained six groups) with consideration to phase match and soldered to a PCB board. The phase matched coils are shown in Figure 3.

Each coil group terminals were connected to a Schottky diode bridge rectifier for DC current conversion. All rectifier outputs in both halves of the stators were serially connected to provide the desired voltage level and to compensate for possible phase misalignment when opposite teeth shifted at some angles. The main parameters (geometrical, mechanical, and electrical) of the proposed AFPM generator are shown in Table 1.

### 2.2. Analytical Estimation of the Effect of Cogging Torque

The cogging effect is manifested due to the attractive forces acting between permanent magnets of a rotor and stator teeth. The cogging effect produces an alternating torque, which disturbs even rotor movement especially perceptible at low velocities, and therefore causes technical difficulties in AFPM machines. In this study, we aimed at finding a relatively simple way to diminish the magnitude cogging effect. This method was based on the optimal angle shifting between opposite teeth of a double stator. To ensure this approach, estimation of the produced interactive forces (torque) between a rotor and stator must be carried out for the different stator–rotor relative dispositions. The cogging torque value can be obtained as the derivative of the total magnetic field energy E in the stator-rotor structure vs. rotating angle α as follows:

(1) $$T=\frac{\partial E}{\partial \alpha }$$

**Figure 4:**
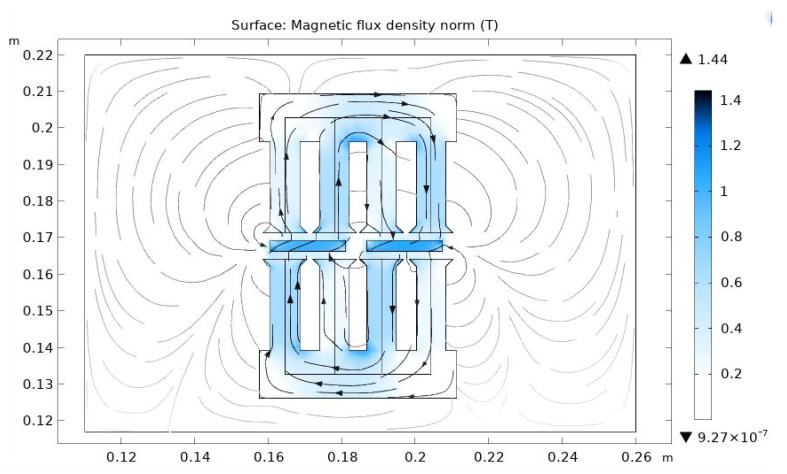
The workspace window for the calculation of a magnetic field.

The total energy is a result of the interaction between stator teeth with all PMs in the rotor. The system of partial differential equations (PDE) must be solved to find the total energy analytically. The theoretical decision of such a PDE system for a complex ferromagnetic stator tooth-PM configuration seems practically impossible. Therefore, we developed a semi-analytical approach based on numerical calculations of magnetic field energy for different rotating angles with the following analytical approximation of obtained values using a polynomial function and its differentiation. The numerical assessment of magnetic field flux and energy was carried out using COMSOL software [31]. The cogging torque value was obtained as the numerical derivative of magnetic field energy in the stator-rotor structure vs. the rotating angle. Considering the periodic change in the disposition between the stator teeth and PMs in the rotor, and therefore the alternating nature of the cogging effect, the calculations were done for the restricted angle variety in a range that was 3–5 times more than the entire period of a cogging torque change. Then, the obtained points of torque-angle pairs were approximated by a spline third-order polynomial function (MATLAB), which numerically calculated the derivative representing the magnitudes of cogging torque. Part of the COMSOL scheme of a solution is represented by the graphical picture in Figure 4.

**Figure 5:**
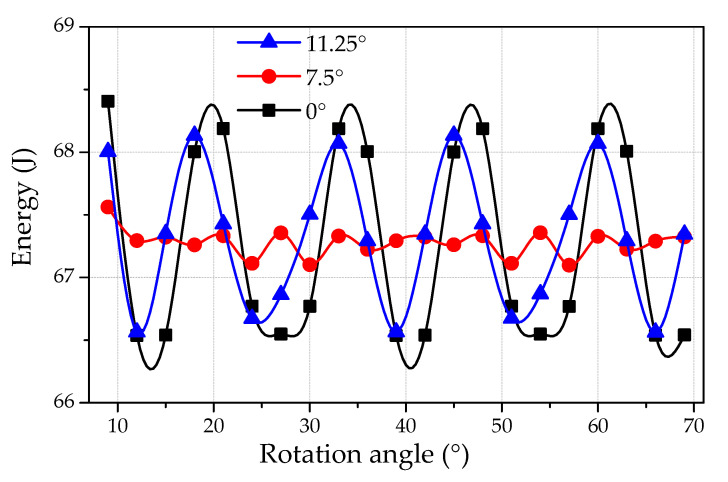
The magnetic field energy vs. the rotating angle for three relative angles between opposite teeth (0°, 7.5°, and 11.25°).

**Figure 6:**
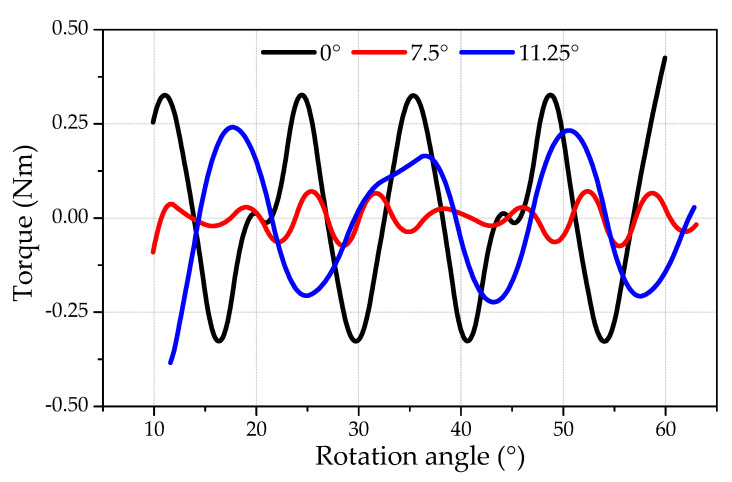
Theoretical prediction of the cogging torque magnitude vs. the rotating angle for three relative angles between opposite teeth (0°, 7.5°, and 11.25°).

Figure 5 represents the magnetic field energy for three relative positions of opposite teeth, whereas Figure 6 shows the corresponding cogging torque. The magnetic energy changes periodically with respect to rotation angle. However, the average magnitude of magnetic energy remains constant for all disposition angles (0°, 7.5°, and 11.25°). Similar behavior is observed for cogging torque (Figure 6). Following the law of energy conservation, the average value of periodically changing magnitude should be equal to zero. This was proven by the calculations and is seen in Figure 6. It can be seen that the cogging torque is minimum for the disposition angle of 7.5°, hence it is the optimal angle. The optimal angle (7.5°) is equal to half of the angle (15°) between two neighboring teeth. At this position, the permanent magnet feels equal attraction influences from both teeth. Thus, the opposite teeth are located exactly opposite the contrary stator teeth intervals. Therefore, the cogging torque effect should be maximally compensated for based on the principle of symmetry. The cogging torque amplitude for the optimal disposition angle (7.5°) is 4–5 lower than for the shifting angle of 0°. Other angles between opposite stator teeth (0° and 11.25°) are not suitable for cogging torque reduction. However, the cogging torque is slightly lower at 11.25° than that of 0°.

### 2.3. Output Characteristics

**Figure 7:**
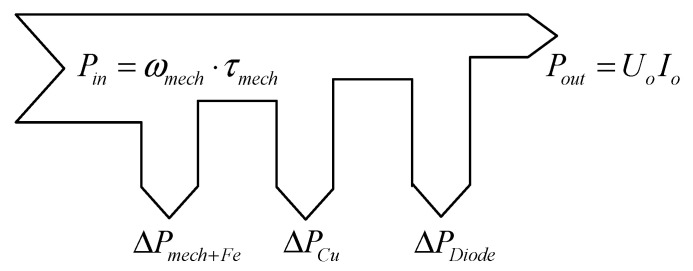
Energy flow diagram of the AFPM generator.

The output characteristics are used to analyze the AFPM generator. These characteristics are output voltage, power, and efficiency as a function of DC load current. These parameters are calculated based on the energy flow, as shown in Figure 7.

Mechanical and iron power losses ($\Delta P_{mech+Fe}$) can be estimated by the multiplication of the special coefficient of mechanical losses ($K_{mech}$) and rotating velocity ($\omega_{mech}$) [32] as follows:

(2) $$\Delta P_{mech+Fe}=K_{mech}\cdot \omega_{mech}$$

The copper losses are taken into consideration as a result of the stator winding resistance ($R_{st}$), coil current ($I_{rms}$), and the total number of stator coils ($N_{c}$), and are calculated as follows:

(3) $$\Delta P_{Cu}=I_{rms}^{2}\cdot R_{st}\cdot N_{c}$$

The AC current is rectified by Schottky diodes to prevent the misaligning of the voltage phase in both stator windings, which helps to diminish diode voltage drop and diode power losses. The diode also helps to create a serial electrical connection between stator windings. Thus, the electrical generator output provides a load DC current. Such a solution allows a relative angular shift between opposite stator teeth and diminishes the cogging torque. However, diode usage in the circuit causes additional power losses, which should be considered to estimate the generator output. These losses ($\Delta P_{diode}$) can be calculated [33] as follows:

(4) $$\Delta P_{diode}=I_{av}\cdot \Delta V_{D}\cdot N_{D}$$

where $I_{av}$ is the average single diode current, $\Delta V_{D}$ is the voltage drop on a single diode estimated as a constant value of ~0.2–0.4 V for the Schottky diode [33], and $N_{D}$ is the number of serially connected diodes in the entire output circuit.

Special attention should be applied to the assessment of the output voltage, which is calculated according to electromotive force (EMF) produced by serially connected stator windings. The EMF of a single coil ($E_{0}$) and of an entire stator winding ($E_{T}$) are estimated based on Faraday law as follows:

(5) $$\begin{array}{l} E_{0}=n_{t}\frac{d\varphi }{dt}=n_{t}\frac{d\varphi }{d\alpha }\frac{d\alpha }{dt}=n_{t}\omega \frac{d\varphi }{d\alpha }\\ E_{T}=N_{c}\cdot E_{0} \end{array}$$

where *α* (rad) and *ω* (rad/s) are the rotation angle and velocity, and *φ* (Wb) is the magnetic flux penetrating every single coil.

**Figure 8:**
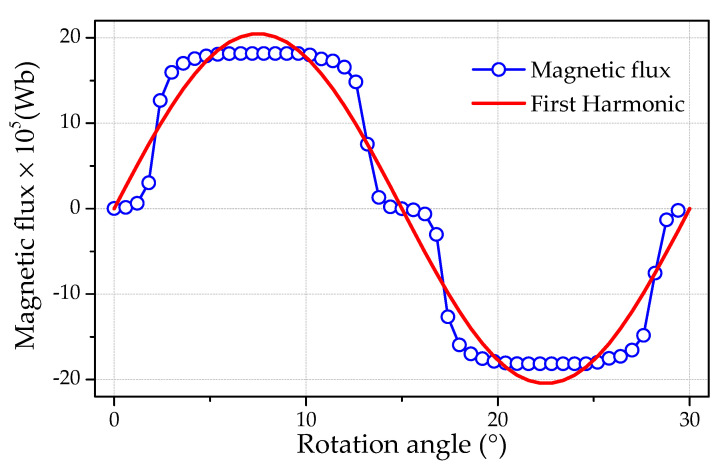
Magnetic flux penetrating a single coil of stator windings as a function of the rotation angle. The solid line represents the first harmonic of magnetic flux.

The magnetic flux derivative was obtained knowing the flux dependence on the rotation angle, which was obtained numerically using COMSOL software [31]. The magnetic flux dependence on rotation angle for the shifting displacement between opposite stator teeth is shown in Figure 8. The derivative of this curve was evaluated with the decomposition of obtained points by a Fourier transform. The analytical differentiation of the first harmonic was produced for simplicity, since its amplitude was significantly superior to others.

The alternating magnetic flux period considering the number of PMs was equal to 30° for all shifting angles. However, amplitudes were different, as shown in Table 2. It can be observed that the cogging torque reduction was accompanied with slightly decreased magnetic flux, which should be considered in the AFPM design. All theoretical calculations mentioned in Section 3 and Section 4 were verified further by experimental investigations.

## 3. Experimental Setup and Methodology

**Figure 9:**
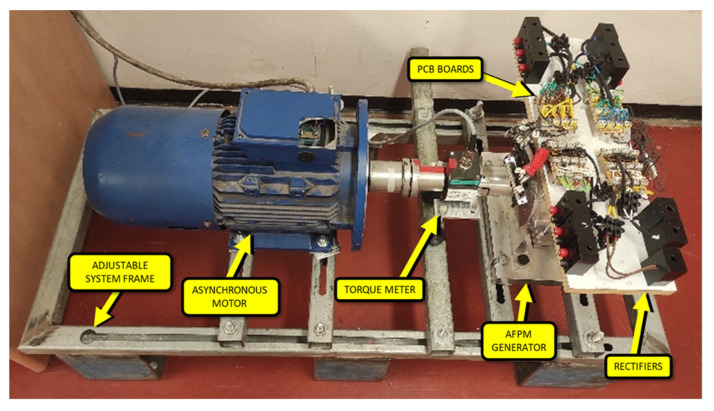
Complete setup of the experimental system.

Figure 9 shows the experimental setup and a special stand which was designed for conducting the experimental tests. The stand included a frame with the installed AFPMG connected to the prime mover through a torque and speed meter. The prime mover was an asynchronous 4-pole motor, 400 V, 3 kW fed by the variable-frequency drive capable of changing velocity in the range of 0–2200 rpm. The torque and speed meter (ATM-6090) had a measurement range of rotational speed 0–3000 rpm and torque 0–20 Nm [34]. The entire system was hinged to a custom-built adjustable system frame, and both machines were precisely leveled to one another (Figure 9).

**Figure 10:**
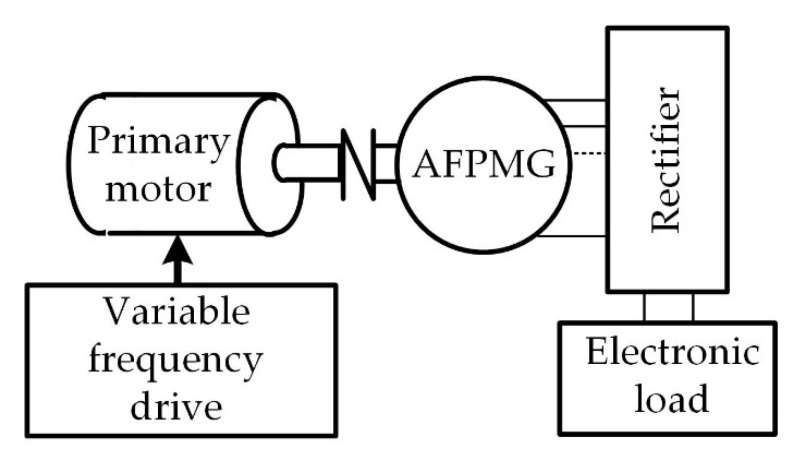
Schematic diagram of the experimental setup.

The experimental tests were conducted according to Figure 10. The experimental tests were as follows: First, the minimum allowable air gap (due to mechanical rotor-stator design) and the angle between opposite teeth of halves of stators were adjusted. The generator was mounted on the workbench (Figure 9). Initially, the output characteristics (*V*, *I*, *τ*, and *n*) of the generator were obtained. The output characteristics included voltage (*V*), current (*I*), torque (*τ*), and velocity (*n*). The primary motor was operated at the required rotating velocity, and the velocity was changed for each set of experiments (1200, 1300, 1400, 1500, 1600, and 1700 rpm). The generator was loaded with an electronic load (M9714B, 0–60 A/0–500 V/1200 W) [35] for each set of experiments. The current was changed (0–2000 mA), and the voltage was measured and recorded accordingly. The real velocity and torque were also measured. Furthermore, the pole-to-pole position of the stators (relative angle between stators) was shifted at different angles (0°, 7.5°, and 11.25°). The cogging torque together with output characteristics were measured for each angle. The cogging effect was evaluated at a very slow speed of generator movement (~0.5 rpm). The instantaneous torque was measured this time, and the pulses obtained from a velocity sensor were recorded. The average cogging was estimated by the instantaneous torque magnitudes and the rotating angle propagation vs. time. The angle circulation was calculated due to the pulses obtained from the sensor of a rotating movement. The single turn of a sensor axis (and therefore of the generator rotor) included 60 pulses. Consequently, each pulse was equal to six angle degrees.

The cogging torque was calculated for the varying rotation angles. The torque sensor generated time-dependent data from the scope. Knowing the distance between pulses allowed us to translate time-dependent to an angle-dependent torque signal. Furthermore, juxtaposing the torque and angle data allowed us to determine the function of the cogging torque vs. the rotation angle. On the basis of this function, the average cogging torque was estimated as follows:

(6) $${\left(\tau_{cog}\right)}_{av}=\frac{1}{\left(\alpha_{2}-\alpha_{1}\right)}\int_{\alpha_{1}}^{\alpha_{2}}\tau \left(\alpha \right)d\alpha$$

The integration was solved numerically by summating the current values of a torque multiplied on the elemental growths of the angle propagation.

## 4. Results and Discussion

**Figure 11:**
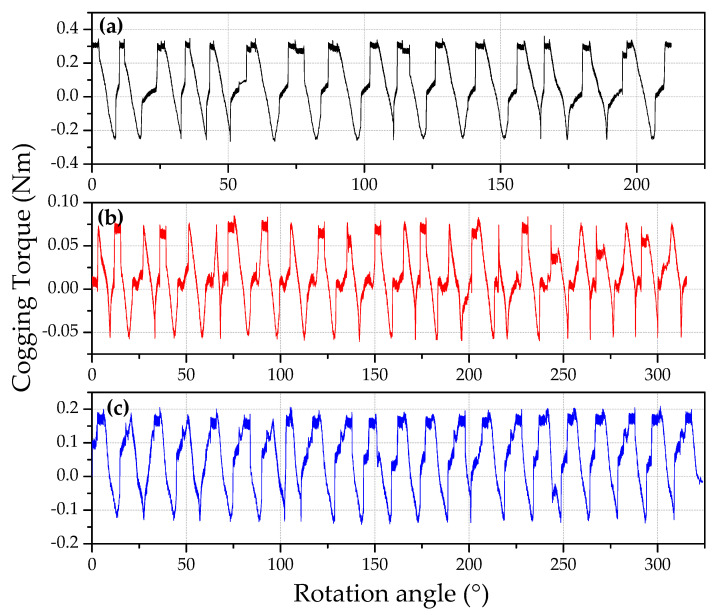
Cogging torque as a function of rotation angles for three relative angles between opposite teeth. (**a**) 0°; (**b**) 7.5°; (**c**) 11.25°.

Cogging torque and the performance of the generator were measured at different angles, i.e., 0°, 7.5°, and 11.25°. The performance of the generator was also checked at various velocities and under varying loads. The cogging torque values were measured using a torque meter. The cogging torque values as a function of rotation angles for different angles (0°, 7.5°, and 11.25°) are shown in Figure 11a–c. It is interesting to note that the maximum value of cogging torque was significantly reduced at the displacement of the stator between 0° to 7.5° and 11.25°. As expected, the frequency of the cogging torque increased with a reduction in magnitude. It is obvious from the graphs that 7.5° and 11.25° are preferred configurations in cogging torque considerations. The cogging torque amplitude and the average value of the cogging torque are the main indicators for the optimal angle between opposite teeth. These parameters are estimated by taking the average of the torque curves in each configuration (Table 3). We found that the cogging torque and average cogging torque were lowest at 7.5° as compared with that of 0° and 11.25°. It is obvious from the average torque and its amplitude that the preferred configuration is 7.5° displacement since there is a significant improvement of nearly 50% in the average torque and four to five times decrease in the torque amplitude. According to the theoretical investigation (Section 2), the average cogging torque magnitude should be equal to zero. However, the presence of radial torque leads to enhancement of the friction force. This circumstance explains the presence of the average cogging torque in experiments.

**Figure 12:**
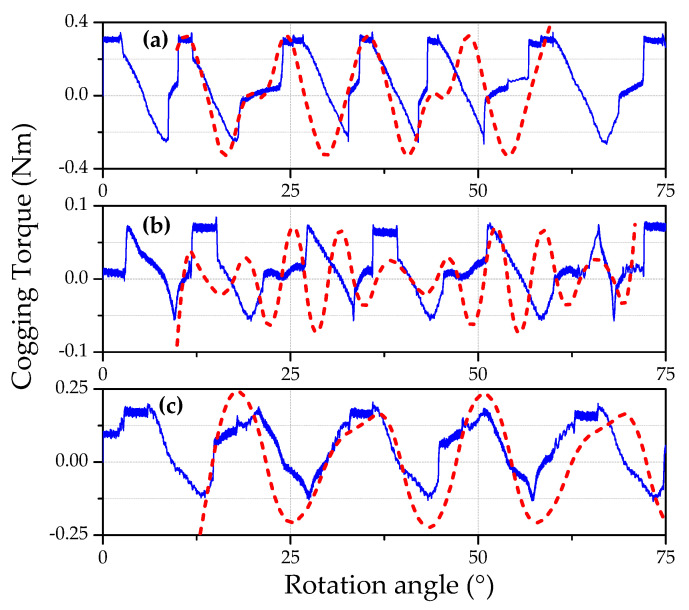
Comparison between analytical and measured cogging torque for three relative angles between opposite teeth. (**a**) 0°; (**b**) 7.5°; (**c**) 11.25°. The solid lines represent the measured cogging torque, and the dashed lines represent the calculated cogging torque.

Figure 12 illustrates the comparison between measured and analytically estimated cogging torque for three relative angles between opposite stator teeth (0°, 7.5°, and 11.25°). The cogging torque is minimum at an optimal angle of 7.5° for both experimental and theoretical analysis. Notably, there is a discrepancy between measured and analytical data, which is minimum for 0° and 11.25° and maximum for 7.5°. These discrepancies can be explained based on manufacturing tolerance and inevitable friction forces. The stator is designed using the electro-erosion technology, which has comparatively low accuracy. Hence, the deviation of teeth width is about 40–50 μm. It is important to point out that these circumstances do not influence the selection of the optimal angle.

**Figure 13:**
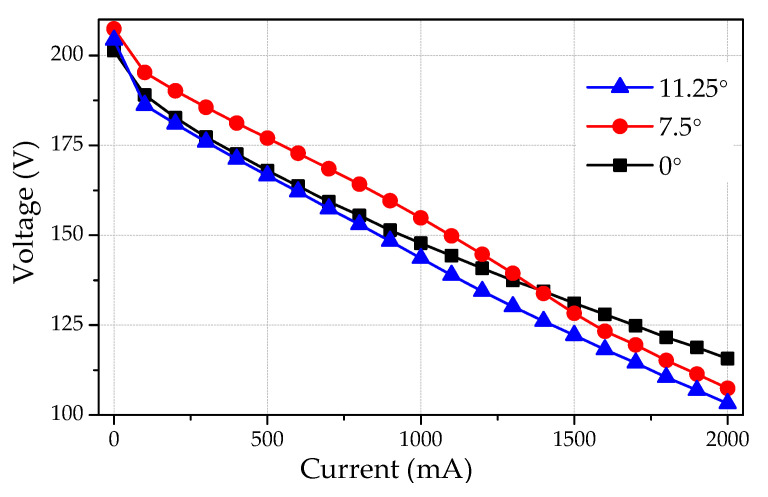
Output voltage vs. load current for three shifting angles (0°, 7.5°, and 11.25°).

**Figure 14:**
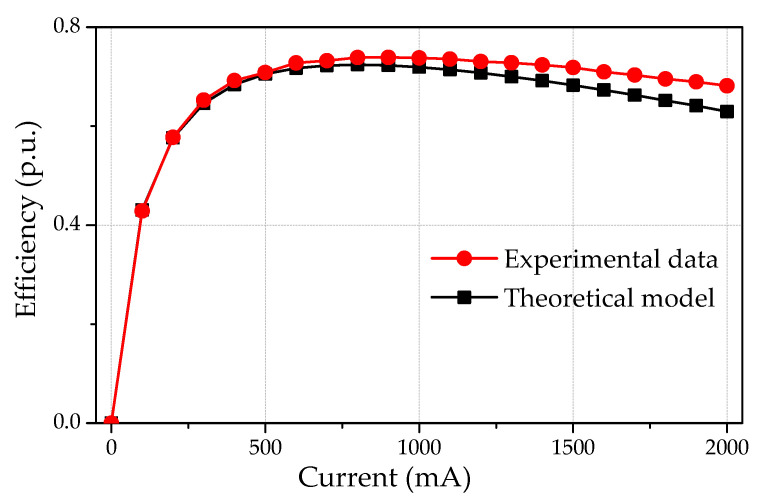
AFPM generator efficiency for *n* = 1500 rpm.

Here, we also want to discuss the performance of the generator at a low cogging torque configuration. This study also verified that any configuration change would not damage the performance capability of the generator. The different configurations (0°, 7.5°, and 11.25°) were tested at a speed range of 1200–1700 rpm and varying loads of 0–2 A. The current-voltage (I–V) curves of the AFPM generator at 1500 rpm are shown in Figure 13. The output voltage remained relatively equal for different angles between opposite stator teeth (0°, 7.5°, and 11.25°). Figure 14 demonstrates the efficiency of the AFPM generator as a function of load current. Both experimental and theoretical results show good agreement.

The efficiency and power of the generator for different shifting angles and rotating velocities are represented in Table 4 and Table 5. Last but not least, the main goal of the research is achieved, and the performance of the machine has improved mechanically and electrically.

## 5. Conclusions

This study investigated cogging torque reduction in AFPM machines using stator to stator angle displacement. The machine topology allowed us to adjust its geometry to investigate and achieve the target. The experimental and theoretical analysis proved that the 7.5° displacement exhibited a significant reduction in cogging torque (four to five times) and good performance. However, shifting to the optimal angle was accompanied by a slight decrease in the magnetic flux and output power (~5–10%). More importantly, it led to improved efficiency at high rotational speeds. This study showed the superiority of AFPM generators over conventional radial flux types regarding the achieved output power versus generator weight. This advantage is especially noticeable at low rotational speeds.

## Figures and Tables

**Table 1:** The main parameters of the proposed AFPM generator.

Parameters	Range
Diameter (mm)	155
Width (mm)	95
Weight (kg)	4.2
Rotational speed range (rpm)	600–2000
Output power (W)	110–320
Output voltage (V)	120–220

**Table 2:** Amplitude of magnetic flux in a single stator coil.

**Shifting Angle (°)**	0	7.5	11.25
**Amplitude × 10^5^ (Wb)**	20.47	16.6	19.45

**Table 3:** Average and standard cogging torque for different angles between opposite teeth.

Angle (°)	Average Torque	Torque
0	0.1865	0.143
7.5	0.0725	0.0737
11.25	0.0978	0.089

**Table 4:** Maximum efficiency at different velocities and angles.

Velocity (rpm)	Maximum Efficiency		
	0°	7.5°	11.25°
1200	0.705	0.749	0.700
1300	0.701	0.759	0.707
1400	0.718	0.767	0.711
1500	0.739	0.759	0.739
1600	0.738	0.757	0.746
1700	0.736	0.769	0.751

**Table 5:** Maximum power at different velocities and angles.

Velocity (rpm)	Maximum Power (W)		
	0°	7.5°	11.25°
1200	146.2	156.3	148.5
1300	186.0	174.4	164.0
1400	207.1	196.3	180.9
1500	230.0	211.7	199.4
1600	248.1	229.4	217.3
1700	269.8	248.1	233.9

## Data Availability

The data presented in this study are available on request from the corresponding author.
